# Supplementary material for: Pyruvate Dehydrogenase Kinase 1 inhibition mediated oxidative phosphorylation enhancement in cartilage promotes osteoarthritis progression
Source: BMC Musculoskelet Disord. 2023 Jul 20;24:597. doi: 10.1186/s12891-023-06585-6 (PMC10357736; doi:10.1186/s12891-023-06585-6)
Supplement: Supplementary file 1 — Additional file 1. [file 12891_2023_6585_MOESM1_ESM.zip › MF.Gsea.1653627316242/GOMF_14_3_3_PROTEIN_BINDING.html]

Details for gene set GOMF\_14\_3\_3\_PROTEIN\_BINDING[GSEA]

|  || Dataset | 55457\_collapsed\_to\_symbols.cls.cls #OA\_versus\_normal.cls.cls #OA\_versus\_normal\_repos |
| Phenotype | cls.cls#OA\_versus\_normal\_repos |
| Upregulated in class | normal |
| GeneSet | GOMF\_14\_3\_3\_PROTEIN\_BINDING |
| Enrichment Score (ES) | -0.51822644 |
| Normalized Enrichment Score (NES) | -1.6884496 |
| Nominal p-value | 0.006779661 |
| FDR q-value | 0.10963567 |
| FWER p-Value | 0.918 |
Table: GSEA Results Summary

  

Fig 1: Enrichment plot: GOMF\_14\_3\_3\_PROTEIN\_BINDING      
 Profile of the Running ES Score & Positions of GeneSet Members on the Rank Ordered List

  

| SYMBOL | TITLE | RANK IN GENE LIST | RANK METRIC SCORE | RUNNING ES | CORE ENRICHMENT || 1 | AKT1 | AKT serine/threonine kinase 1 [Source:HGNC Symbol;Acc:HGNC:391] | 1192 | 0.478 | -0.0479 | No |
| 2 | TBC1D22A | TBC1 domain family member 22A [Source:HGNC Symbol;Acc:HGNC:1309] | 1718 | 0.395 | -0.0517 | No |
| 3 | KLHL22 | kelch like family member 22 [Source:HGNC Symbol;Acc:HGNC:25888] | 1982 | 0.363 | -0.0382 | No |
| 4 | RBM7 | RNA binding motif protein 7 [Source:HGNC Symbol;Acc:HGNC:9904] | 2620 | 0.288 | -0.0607 | No |
| 5 | PRKCZ | protein kinase C zeta [Source:HGNC Symbol;Acc:HGNC:9412] | 2760 | 0.274 | -0.0459 | No |
| 6 | AANAT | aralkylamine N-acetyltransferase [Source:HGNC Symbol;Acc:HGNC:19] | 3017 | 0.248 | -0.0426 | No |
| 7 | BAD | BCL2 associated agonist of cell death [Source:HGNC Symbol;Acc:HGNC:936] | 3788 | 0.178 | -0.0858 | No |
| 8 | PI4KB | phosphatidylinositol 4-kinase beta [Source:HGNC Symbol;Acc:HGNC:8984] | 4485 | 0.114 | -0.1292 | No |
| 9 | KIF13B | kinesin family member 13B [Source:HGNC Symbol;Acc:HGNC:14405] | 5448 | 0.037 | -0.2004 | No |
| 10 | PRKCE | protein kinase C epsilon [Source:HGNC Symbol;Acc:HGNC:9401] | 5926 | 0.000 | -0.2374 | No |
| 11 | NEK1 | NIMA related kinase 1 [Source:HGNC Symbol;Acc:HGNC:7744] | 6266 | -0.025 | -0.2614 | No |
| 12 | HLA-F | "major histocompatibility complex, class I, F [Source:HGNC Symbol;Acc:HGNC:4963]" | 7196 | -0.096 | -0.3246 | No |
| 13 | ARRB2 | arrestin beta 2 [Source:HGNC Symbol;Acc:HGNC:712] | 7255 | -0.100 | -0.3198 | No |
| 14 | RIPOR1 | RHO family interacting cell polarization regulator 1 [Source:HGNC Symbol;Acc:HGNC:25836] | 8250 | -0.175 | -0.3807 | No |
| 15 | TBC1D22B | TBC1 domain family member 22B [Source:HGNC Symbol;Acc:HGNC:21602] | 8571 | -0.199 | -0.3869 | No |
| 16 | KSR1 | kinase suppressor of ras 1 [Source:HGNC Symbol;Acc:HGNC:6465] | 8774 | -0.214 | -0.3825 | No |
| 17 | SRPK2 | SRSF protein kinase 2 [Source:HGNC Symbol;Acc:HGNC:11306] | 10522 | -0.376 | -0.4831 | Yes |
| 18 | RIPOR2 | RHO family interacting cell polarization regulator 2 [Source:HGNC Symbol;Acc:HGNC:13872] | 10698 | -0.393 | -0.4600 | Yes |
| 19 | DDIT4 | DNA damage inducible transcript 4 [Source:HGNC Symbol;Acc:HGNC:24944] | 10818 | -0.407 | -0.4311 | Yes |
| 20 | PPP1R12A | protein phosphatase 1 regulatory subunit 12A [Source:HGNC Symbol;Acc:HGNC:7618] | 11259 | -0.465 | -0.4218 | Yes |
| 21 | IRS2 | insulin receptor substrate 2 [Source:HGNC Symbol;Acc:HGNC:6126] | 11390 | -0.485 | -0.3866 | Yes |
| 22 | SYNPO2 | synaptopodin 2 [Source:HGNC Symbol;Acc:HGNC:17732] | 12688 | -0.855 | -0.4074 | Yes |
| 23 | ZFP36 | ZFP36 ring finger protein [Source:HGNC Symbol;Acc:HGNC:12862] | 12846 | -1.052 | -0.3213 | Yes |
| 24 | SIK1 | salt inducible kinase 1 [Source:HGNC Symbol;Acc:HGNC:11142] | 12863 | -1.104 | -0.2193 | Yes |
| 25 | HDAC7 | histone deacetylase 7 [Source:HGNC Symbol;Acc:HGNC:14067] | 12870 | -1.131 | -0.1140 | Yes |
| 26 | ZFP36L1 | ZFP36 ring finger protein like 1 [Source:HGNC Symbol;Acc:HGNC:1107] | 12888 | -1.244 | 0.0009 | Yes |
Table: GSEA details [plain text format]

  

Fig 2: GOMF\_14\_3\_3\_PROTEIN\_BINDING      
 Blue-Pink O' Gram in the Space of the Analyzed GeneSet

  

Fig 3: GOMF\_14\_3\_3\_PROTEIN\_BINDING: Random ES distribution      
 Gene set null distribution of ES for **GOMF\_14\_3\_3\_PROTEIN\_BINDING**
